# The RNA export factor TbMex67 connects transcription and RNA export in *Trypanosoma brucei* and sets boundaries for RNA polymerase I

**DOI:** 10.1093/nar/gkad251

**Published:** 2023-04-18

**Authors:** Berta Pozzi, Arunasalam Naguleswaran, Francesca Florini, Zahra Rezaei, Isabel Roditi

**Affiliations:** Institute of Cell Biology, University of Bern, Bern, Switzerland; Institute of Cell Biology, University of Bern, Bern, Switzerland; Institute of Cell Biology, University of Bern, Bern, Switzerland; Professor Alborzi Clinical Microbiology Research Center, Shiraz University of Medical Sciences, Shiraz, Iran; Institute of Cell Biology, University of Bern, Bern, Switzerland

## Abstract

TbMex67 is the major mRNA export factor known to date in trypanosomes, forming part of the docking platform within the nuclear pore. To explore its role in co-transcriptional mRNA export, recently reported in *Trypanosoma brucei*, pulse labelling of nascent RNAs with 5-ethynyl uridine (5-EU) was performed with cells depleted of TbMex67 and complemented with a dominant-negative mutant (TbMex67-DN). RNA polymerase (Pol) II transcription was unaffected, but the procyclin loci, which encode mRNAs transcribed by Pol I from internal sites on chromosomes 6 and 10, showed increased levels of 5-EU incorporation. This was due to Pol I readthrough transcription, which proceeded beyond the procyclin and procyclin-associated genes up to the Pol II transcription start site on the opposite strand. Complementation by TbMex67-DN also increased Pol I-dependent formation of R-loops and γ-histone 2A foci. The DN mutant exhibited reduced nuclear localisation and binding to chromatin compared to wild-type TbMex67. Together with its interaction with chromatin remodelling factor TbRRM1 and Pol II, and transcription-dependent association of Pol II with nucleoporins, our findings support a role for TbMex67 in connecting transcription and export in *T. brucei*. In addition, TbMex67 stalls readthrough by Pol I in specific contexts, thereby limiting R-loop formation and replication stress.

## INTRODUCTION

*Trypanosoma brucei* is an early branching unicellular eukaryote and the parasite responsible for human and animal trypanosomiasis in sub-Saharan Africa. This pathogen goes through a very complex life cycle between its insect (tsetse fly) and mammalian hosts, accompanied by unusual features in terms of gene expression. With few exceptions, protein-coding genes are organised in polycistronic transcription units (PTUs) preceded by a single transcription start site (1). Polycistronic precursor RNAs are processed into monocistronic mRNAs by coupled trans-splicing and polyadenylation so that all mRNAs have the same 39 base spliced leader at their 5′ ends (2–4). Steady state levels of mRNAs derived from the same precursor can vary by more than two orders of magnitude, owing to intrinsic differences in their stability (5–10). In contrast, pulse labelling of isolated nuclei with radioactive precursors showed much more uniform labelling of nascent transcripts of genes within the same transcription unit, as would be expected if they were part of the same precursor (11–13). These early studies were restricted to a few regions of the genome and could not be performed with intact cells. Recently, however, we established global run-on sequencing (GRO-seq) for live trypanosomes (14).

Most protein-coding genes in *T. brucei* are transcribed by RNA polymerase II (Pol II), but some developmentally regulated genes are transcribed by RNA polymerase I (Pol I). This is the case for the procyclins and variant surface glycoproteins (VSG), the surface proteins expressed by procyclic forms and bloodstream forms in their insect and mammalian hosts, respectively (15). Although trypanosomes encode approximately two thousand VSG genes, only one VSG is expressed at a time in the mammalian host (reviewed in (16–18)). The active VSG is always the last gene in a telomeric PTU that also contains multiple expression-site associated genes (19–21). This locus forms a distinct subnuclear compartment, known as the Expression Site Body (ESB), that recruits RNA Pol I (22) and is associated with a spliced leader array body, presumably to facilitate high expression of the VSG (23,24). By contrast, procyclin genes (EP1, EP2, EP3 and GPEET) occur at internal positions on chromosomes 6 and 10. These PTUs are relatively short, usually consisting of two procyclin genes followed by up to four procyclin-associated genes (PAGs) (25,26). In common with the ribosomal DNA loci, which are also transcribed by Pol I, all procyclin genes are localised at the nucleolar periphery in procyclic forms (22).

The genome organisation and relative paucity of Pol II promoters (1,27) has meant that most studies of gene expression in trypanosomes have focused on post-transcriptional control, principally mRNA stability, maturation by splicing and polyadenylation, and mRNA export (reviewed in (28)). It is generally assumed that Pol II transcription initiation and elongation in *T. brucei* are unregulated. However, there are specific chromatin marks at the boundaries of the PTUs. Modified histones H3K4me3 and H4K10ac, and histone variants H2AZ and H2BV map to open chromatin at the transcription start sites, while H3V and H4V map to compact chromatin at transcription termination sites (1,29). This indicates that trypanosome PTUs could be analogous to other eukaryotic transcription units and that Pol II transcription might be regulated to a certain degree.

The extent to which the multiple steps of RNA metabolism are orchestrated has not been as thoroughly investigated in trypanosomes as in other eukaryotes (30). It was recently proposed that trypanosomes can initiate nuclear export co-transcriptionally, based on the detection of uncapped, unspliced mRNAs on the cytoplasmic side of the nuclear pore by single molecule RNA fluorescence *in situ* hybridisation (smFISH) (31). The nuclear pore architecture of *T. brucei* supports this notion since, unlike other eukaryotes, its nucleoporin composition and distribution are symmetrical, and this might account for less strict regulation and quality control of exported transcripts (32). In general, RNA export might be more promiscuous in trypanosomes than in other organisms. TbMex67 (Tb927.11.2370) is the major mRNA export factor known to date in trypanosomes; together with its smaller partner TbMtr2 and the TbNup76 complex, it forms part of the mRNA export docking platform within the nuclear pore with Ran and other putative Ran binding proteins (32).

Recently, it was proposed that two other proteins in *T. brucei*, designated Mex67b (Tb927.11.2340) and Mex67L (Tb927.10.2060) are Mex67 paralogs, based on their interaction with TbMtr2 and the presence of LRR and NTF2-like domains (https://www.biorxiv.org/content/10.1101/2022.06.27.497849v1). The genes encoding them were too divergent to be identified as relatives of TbMex67 by BLASTN searches. Mex67L was described as having a non-canonical function as RNAi did not cause retention of polyA RNA in the nucleus. RNAi against Mex67b did cause polyA retention, but had no effect on growth of procyclic forms, suggesting that it might affect a non-essential class of RNAs. This contrasts with depletion of TbMex67 which results in both polyA RNA retention and a growth phenotype (33).

TbMex67 possesses a CCCH-type zinc finger at its N-terminus, which is essential for its function (33,34). Moreover, deletion of this domain transforms the protein into a dominant negative mutant that blocks RNA export even in the presence of endogenous wild-type TbMex67 (33). The TbMex67/Mtr2 complex has additional functions in tRNA export (35) and in ribosome biogenesis (36,37), a task that is shared with the rRNA transporters TbXPO1 and TbNMD3. The latter have also been shown to be mRNA export factors (38). It is an open question whether the TbMex67/Mtr2 and TbNMD3/XPO1 export pathways interact with or depend on each other, if they carry distinct mRNA cargos, or possibly feedback on transcription. Recently, it was reported that DRBD18, an essential and abundant RNA binding protein of *T. brucei*, associates with TbMex67/Mtr2 *in vivo*, and that DRBD18 downregulation results in partial accumulation of polyadenylated mRNAs in the nucleus (39).

Here, we show that knockdown of TbMex67, and complementation with the dominant negative form lacking the N-terminal zinc finger, increased the transcription of PAGs and downstream regions on chromosomes 6 and 10. Chromatin immunoprecipitation (ChIP)-seq revealed that this was the result of Pol I readthrough and concomitant nucleosome depletion. Furthermore, both Pol I subnuclear localisation and nucleolar organisation were affected. Together, these phenomena suggested a role for TbMex67 in connecting the transcription and export machineries. Consistent with this, we verified that TbMex67 binds chromatin at actively transcribed genes, assisted by its zinc finger motif, and that the truncated form loses its chromatin-binding properties and nuclear localisation.

## MATERIALS AND METHODS

### *Trypanosoma brucei* strains and plasmids

Procyclic forms of *T. brucei brucei* Lister 29–13 (40) and derivatives used in this study were cultivated in SDM79 (41) supplemented with 10% foetal bovine serum. The TbNMD3 RNAi cell line has been described previously (38). The TbMex67 RNAi cell line and derivatives expressing the dominant-negative mutant (DN) or the zinc finger domain mutant (C29R), and the PTP-tagged TbMex67cell line, all generated by (33) were kindly provided by Bernd Schimanski (University of Bern). TbMex67 is encoded by Tb927.11.2370. TbMex67dNES was obtained by DpnI-mediated mutagenesis of the wild-type TbMex67 plasmid (33) with Phusion polymerase (NEB). The TbRNaseH1 open reading frame (Tb427.07.4930) was amplified by PCR and cloned into pLEW111 with a puromycin resistance cassette. Transfections were performed as previously described and transfectants were selected in the presence of 10 μg ml^−1^ blasticidin, 1 μg ml^−1^ puromycin or 500 μg ml^−1^ nourseothricin (42). Expression was induced by addition of 1 μg ml^−1^ tetracycline to the culture medium. To generate the TbRNaseH1 knockout, parasites were transiently transfected with pAi1C9, a vector encoding T7 polymerase and Cas9 and 24 h later with the appropriate PCR products consisting of the resistance genes flanked by homology regions, as previously described (43,44). All oligonucleotides employed in this study are listed in Supplementary Table S3.

Procyclic forms of EATRO1125 (AnTaR1; (45,46)), cultivated in the same medium, were used for pulldowns of RPB1 and colocalization with NUP1 by fluorescence microscopy.

### Labelling of nascent RNAs

Procyclic forms at a density of 5 × 10^6^ ml^−1^ were pulsed with 200 μm 5-ethynyl uridine (5-EU) for 10 min. Nuclear RNA isolation and copper-dependent click-it chemistry were performed as described (14). cDNA library preparation using an Illumina Truseq RNA sample preparation kit and sequencing with the NextSeq system were performed at Fasteris (Geneva).

### Fluorescence *in situ* hybridization (FISH) and immunofluorescence

RNA FISH was performed according to Cassola and colleagues (47): cells were permeabilized and simultaneously blocked for 30 min in PBS/0.5% saponin/2% BSA, and pre-hybridised for 1 h in hybridisation solution (2% BSA, 5× Denhardt′s solution, 4× SSC, 5% dextran sulphate, 35% formamide, 0.5 μg μl^−1^ tRNA). Cy3-labelled oligo-d(T)_20_ (2 ng μl^−1^ in hybridisation solution) was incubated overnight at room temperature. Cells were then washed with decreasing concentrations of SSC (from 4 × to 1×), and nuclei were stained with Hoechst 33342. Images were captured with a Leica DM 5500 B microscope and analysed using LAS AF software (Leica) and Fiji.

For immunofluorescence, cells were harvested by centrifugation, washed once in PBS, fixed in 4% paraformaldehyde in PBS followed by 10 min incubation with 50 mM NH_4_Cl and permeabilised with Triton X-100 (0.1% in PBS). After blocking in PBS/3% BSA, cells were incubated with primary antibody (1:100 for anti-Pol I (22), L1C6 (48,49), anti-TbMex67 (33), anti-TbγH2A (50)) for 1 h at room temperature, washed 3 times in PBS and subsequently incubated with secondary antibody (1:2000, Alexa Fluor 488 or Cy3 anti-rabbit, mouse or rat, ThermoFisher) for 1 h at room temperature (RT). Cells were then washed 3 times in PBS and stained with Hoechst 33342 prior to embedding in Mowiol (Sigma Aldrich). For RPB1 and NUP1 immunofluorescence, cells were fixed with 1% paraformaldehyde in PBS for 20 min, permeabilized with Triton X-100 (0.1% in PBS) for 10 min, blocked with 3% BSA in PBS for 1 h at RT and overnight incubation with primary antibodies at 4ºC. Subsequent incubation with secondary antibodies and DNA staining were performed as previously described. For S9.6 staining, cells were fixed and permeabilised in ice-cold methanol for 5 min, while all incubations and washes were performed in the presence of 0.1% Triton X-100. S9.6 was employed as primary antibody at a dilution of 1:500 in PBS/3% BSA. Anti-mouse Alexa 488 (Thermo Fisher) was used as secondary antibody at a dilution of 1:2000. When indicated, drug treatment was performed as follows: Actinomycin D (5 μg ml−1, 2 h), alpha-amanitin (100 μg ml−1, 16 h) or CX5461 (3μM, 3 h), for the corresponding time prior to fixation. In the case of RNase H treatment, fixed-permeabilised samples were treated with 2 units of recombinant RNase H (NEB #0297) at 37°C for 20 min. Images were captured with a Leica DM 5500 B microscope and Leica SP8 confocal microscope and analysed using Fiji and CellProfiler (https://cellprofiler.org). All antibodies employed in this study are listed in Supplementary Table S4.

### Immunoprecipitations (IP)

HA-RRM1 and TbMex67-PTP protein complexes were isolated as previously described (33,51). Briefly, procyclic forms were harvested at a density of 5 × 10^6^ ml^−1^ and incubated with 0.1% paraformaldehyde (w/v) in PBS for 8 min at room temperature to cross‐link protein complexes. The reaction was stopped by adding glycine to a final concentration of 125 mM and incubating for 5 min. Cells were washed in PBS and resuspended in lysis buffer (20 mM Tris pH 7.5, 140 mM KCl, 1.8 mM MgCl_2_, 0.1% NP40, 10% glycerol) containing protease inhibitor cocktail (EDTA-free, Roche). Cells were lysed by sonication using a Branson Digital Sonifier three times 10 s, 30 s interval on ice at 10% power. Two percent of the soluble supernatant was reserved as the input fraction and the remainder was used for immunoprecipitation using anti-HA magnetic beads (Pierce™) or IgG-coated Sepharose beads (Roche). In all cases, an untagged culture was used in parallel as a negative control. Protein cross-linking was reversed by incubation of the sample in Laemmli Buffer for 20 min at 95°C. Immunoprecipitated proteins were identified by western blot analysis.

For Western blot analysis, protein samples (immunoprecipitation inputs and eluates or 2 × 10^6^ cell equivalents per lane) were separated on 12% SDS-polyacrylamide gels and transferred to Immobilon-P membrane (Millipore). Membranes were incubated overnight with primary antibodies: anti-TbMex67 (rabbit, 1:2000), anti-RPB1 (rabbit, 1:2000), anti-Pol I (anti-RPA1, rabbit, 1:1000), anti-histone H3 (rabbit, 1:5000) anti-HA 3F10 (rat, 1:2500, Sigma-Aldrich), anti-RRM1 (rabbit, 1:5000) and anti-EIF1α (Mouse, 1:10 000). After washing, membranes were incubated with IRDye^®^ 800CW or 680RD (LI-COR Biosciences) secondary antibodies and detected using an Odyssey imaging system (LI-COR Biosciences). All antibodies used in this study are listed in Supplementary Table S4.

For RBP1 immunoprecipitations and proteomic analyses, IPs were performed as before but using pre-bound anti-RBP1 beads, where antibody was attached to Protein A magnetic beads (Thermo Fisher) by a 2-hour incubation with antibody diluted in lysis buffer (see Supplementary Table S4). Eluates were loaded on an SDS-polyacrylamide gel and run until they had just entered the 12% resolving gel. Proteins were then gel-extracted, subjected to trypsin digestion and analysed by LC–tandem MS. Protein identification was performed at the Proteomics and Mass Spectrometry facility at University of Bern, Switzerland.

### Chromatin immunoprecipitation (ChIP)

ChIP experiments were performed as described previously (14) with minor modifications. 10^8^ parasites were washed once in PBS and fixed with 1% paraformaldehyde (w/v) in PBS for 8 min at room temperature; the reaction was stopped by adding glycine to a final concentration of 125 mM for 5 min. Cells were then washed once in PBS and resuspended in lysis buffer (50 mMTris-HCl, pH 8, 10 mM EDTA, 1% SDS and EDTA-free protease inhibitor cocktail (Roche)). Subsequently, the lysate was sonicated with a Bioruptor (Diagenode) using the following settings: Power ‘L’, ON/OFF (Sec) 30/90 for 7 min, to produce fragments <1 kb. The soluble chromatin was diluted 1:100 in RIPA buffer (10 mM Tris-HCl, pH 7.5, 1 mM EDTA, 0.5 mM EGTA, 1% Triton X-100, 0.1% SDS, 0.1% sodium deoxycholate, 140 mM NaCl) and used for immunoprecipitation. Anti-histone H3 (Abcam ab1791; 2 μg /IP), anti-RPA1 (anti-Pol I; 5 μg/IP) and anti-RPB1 (anti-Pol II; 5 μg /IP) antibodies immobilized on Dynabeads (Thermo Fisher) were used for immunoprecipitation (Supplementary Table S4). TbMex67 ChIP was performed in the same way but with UV crosslinking (3 pulses of auto-crosslink in a Stratalinker UV Crosslinker, Stratagene), instead of paraformaldehyde cross-linking. S9.6 DRIP was carried out, without crosslinking, from chromatin sonicated lysates, as described (52). IPs were performed with pre-bound S9.6 beads (1 μg/IP, Supplementary Table S4). DNA was eluted from the beads by digestion with Proteinase K (50 μg /ml) at 68°C for 2 h, purified by phenol–chloroform extraction and ethanol precipitation, and quantified using a Qubit dsDNA HS Assay kit (Invitrogen). Illumina sequencing was performed at Fasteris (Geneva) using the NextSeq system.

### Sequencing and bioinformatics

Illumina reads were mapped and analysed using the Galaxy platform, as described (14,53,54). ChIP-seq reads were mapped against release 8.1 of the *T. b. brucei* TREU927 genome downloaded from TritrypDB (http://tritrypdb.org/tritrypdb/). The Bowtie2 tool with default parameters was launched in single-end mode with respect to the replicate sequencing libraries. The output was sorted and indexed using SAMtools. Peak calling was performed using MACS2 software using the replicates for each run. Reads from duplicate controls (DNA extracted from the same chromatin samples used for ChIP) were used as input.

Pairwise Spearman correlations were computed using the R package ‘ggplot2’. To generate the profiles heatmaps, alignment coverage was normalised to input coverage using deepTools with the option bamCompare. The normalised alignment as well as the peak enriched intervals (narrow peaks outputs) were used as input files computeMatrix and plotHeatmap. Raw read files are deposited at the European Nucleotide Archives (ENA) http://www.ebi.ac.uk/ena as study PRJEB57072.

### Reagents

Phusion polymerase (NEB M0530S); Bam HI (NEB R0136S), Dpn I (NEB R0176S) and Hind III (NEB R0104S); Ampicilin (Sigma-Aldrich), Blasticidin (Sigma-Aldrich), Nourseothricin (Jena Biosciences), Puromycin (Sigma-Aldrich); Immobilon-P membrane (Millipore); Dynabeads (Thermo Fisher); anti-histone H3 (Abcam ab1791); S9.6 (Kerafast); anti-HA 3F10 (Sigma-Aldrich); IRDye^®^ 800CW or 680RD (LI-COR Biosciences) secondary antibodies; protease inhibitor cocktail (EDTA-free, Roche); Stratalinker UV Crosslinker (Stratagene), Bioruptor (Diagenode); Qubit dsDNA HS Assay kit (Invitrogen); Odyssey imaging system (LI-COR Biosciences). Secondary antibodies (Thermo Fisher).

### Biological resources

*T. brucei brucei* Lister 427 29.13, *E. coli* TG1 competent cells, pLEW100 and pLEW101 vectors (https://www.beiresources.org/Catalog/BEIParasiticProtozoa/NR-42012.aspx, https://tryps.rockefeller.edu/trypsru2_cell_lines.html, https://tryps.rockefeller.edu/trypsru2_plasmids.html).

### Statistical analyses

Imaging quantification was performed with Cell profiler (https://cellprofiler.org/) after developing a suitable pipeline and statistical analysis with Mann-Whitney U-test by Prism v.6 (https://www.graphpad.com/scientific-software/prism/).

### Web sites/data base referencing

TritrypDB, for *T. b. brucei* TREU927 genome (http://tritrypdb.org/tritrypdb/). LeishGEdit (http://www.leishgedit.net/). Galaxy platform (https://usegalaxy.org/). Cell profiler (https://cellprofiler.org/). Prism v.6 (https://www.graphpad.com/scientific-software/prism/).

## RESULTS

### Blocking RNA export has little effect on global patterns of nascent transcription, but increases the transcription of procyclin-associated genes and downstream regions

**Figure 1.**
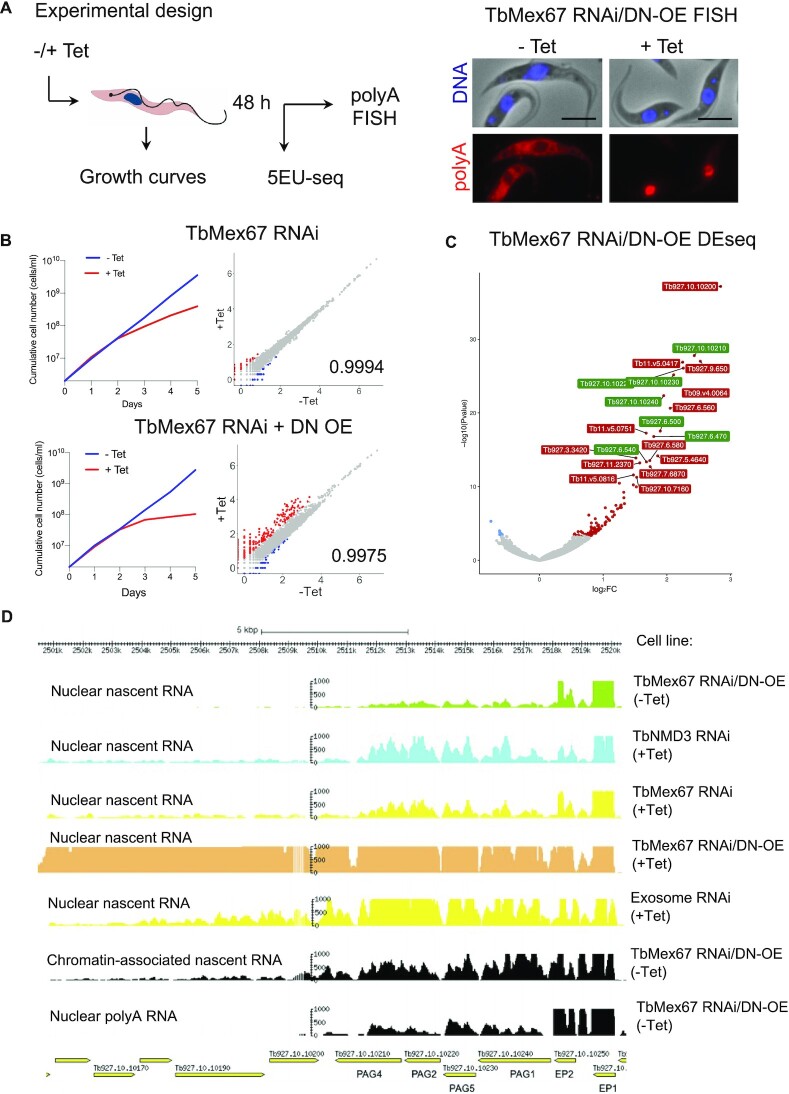
Blocking RNA export has little effect on global patterns of nascent transcription, but increases the expression of procyclin-associated genes and downstream transcripts. (**A**) Scheme of the experimental approach: RNAi was induced by addition of tetracycline (+Tet) and 48 h later the block in export was monitored by polyA RNA FISH. Following a 10 min pulse with 5-EU, nascent RNA was extracted for sequencing. In the case of TbMex67, an RNAi cell line that also overexpressed a dominant negative (DN) isoform upon Tet addition was employed as well (TbMex67 RNAi/DN-OE). Representative pictures for FISH experiments from uninduced and induced TbMex67 RNAi/DN-OE are shown (scale bar, 5 μm). (**B**) Graphs represent growth curves of induced (+Tet) and non-induced (-Tet) RNAi cell lines (left panels) and RPM (reads per million) counts from nascent RNAs with Pearson correlation coefficients (right panels). (**C**) Differential expression analysis (DE-Seq) from nascent transcripts for induced and non-induced TbMex67 RNAi/DN-OE (*n* = 3). Gene IDs for PAGs (procyclin-associated genes) and GRESAGs (gene related to expression site-associated gene) from chromosomes 10 and 6 are indicated in green. Genes outside the procyclin loci are indicated in red. (**D**) Genome browser tracks showing RPKM (reads per kilobase per million) for nascent transcripts from induced (+Tet) and non-induced (–Tet) RNAi cell lines are compared to tracks from chromatin bound RNA and nuclear polyA RNA (from uninduced cells) in a region of Chromosome 10 that encodes for procyclins (EP1 and EP2) and downstream procyclin-associated genes (PAGs).

**Figure 2.**
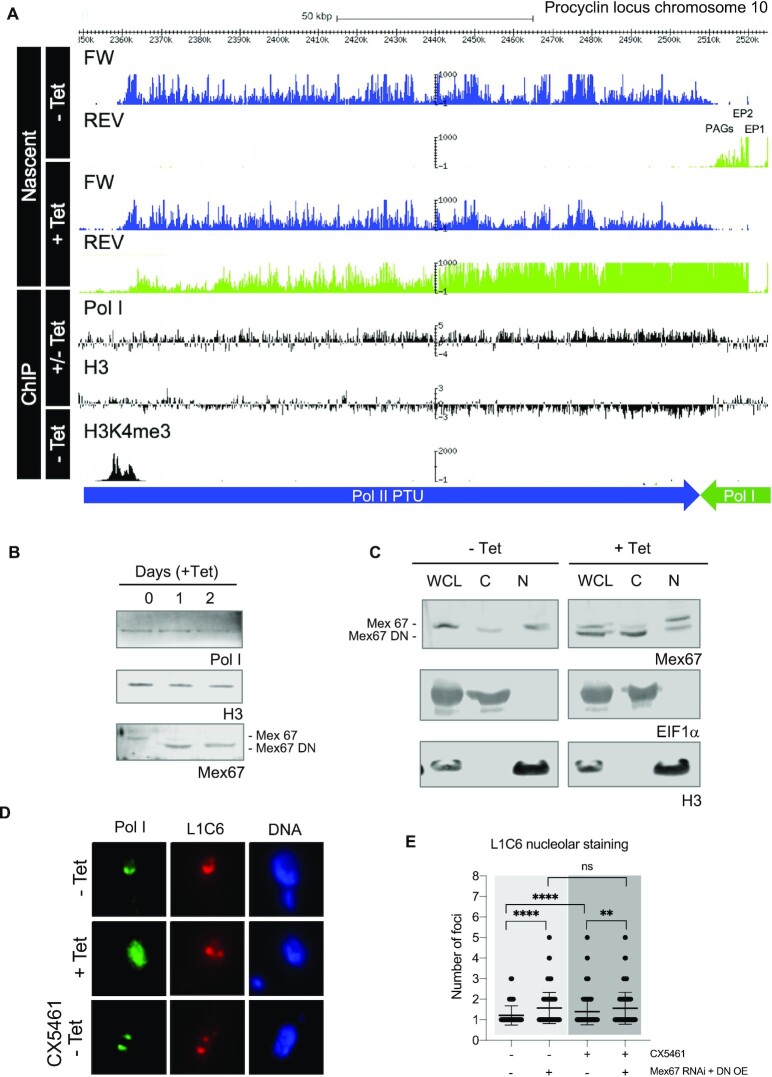
Overexpression of TbMex67 DN results in polymerase I readthrough. (**A**) Patterns of nascent transcription and Pol I and H3 ChIP-seq mapped reads visualized in Genome Browser for a region on chromosome 10 where procyclins and PAGs are encoded. For the nascent transcripts, reads per kb per million (RPKM) were split for the forward (FW) and reverse (REV) strands. Reads for induced (+Tet) and non-induced (–Tet) TbMex67 RNAi/DN-OE are shown. For ChIP-seq of Pol I and H3, the ratios of induced to non-induced (+/– Tet) reads are shown on a log_2_ scale. The track for H3K4me3 (trimethylation of lysine 4 of histone 3) ChIP-seq marks transcription start sites (29). The green tracks and arrow show the directionality of the Pol I polycistronic transcription unit (PTU) while the blue tracks and arrow show the directionality of the Pol II PTU. (**B** and **C**) Western blot for whole cell lysates (left) and subcellular fractionation (right), WCL: whole cell lysate, C: cytoplasm, N: nucleus) from induced and uninduced cells. The antibodies that were used are indicated below each panel. (**D**) Fluorescence microscopy for detection of L1C6 and Pol I from induced and uninduced cells. (**E**) Quantification of the number of L1C6-stained-nucleolar foci per nucleus upon induction of TbMex67 RNAi/DN-OE and/or addition of 3 μM CX5461, an inhibitor of Pol I. Average values are shown with standard deviations and p-values, determined using a Mann–Whitney *U* test. Significant *P*-values are indicated by the asterisks above the graphs (****P< 0.0001; ***P< 0.001; **P< 0.01; *P< 0.05).

Previous sequencing of nascent transcripts from our laboratory (14) showed that neighbouring genes within the same PTU did not always display similar numbers of reads per million per kilobase, as might be expected if they were part of the same precursor RNA. To assess whether this was the result of differential export, we performed RNAi against the general RNA export factor TbMex67, followed by 10 min incubation of intact cells with 5-ethynyl uridine (5-EU) to label nascent transcripts (Figure 1A). We also employed a cell line (TbMex67 RNAi/DN-OE) previously described by Dostalova *et al.* (2013). Upon addition of tetracycline (Tet), this cell line down-regulates TbMex67 by RNAi and overexpresses a dominant negative form lacking the N-terminal CCCH zinc finger domain. Growth curves showed that Tet-induced and -uninduced cultures grew at the same rate for the first two days (Figure 1B). Nevertheless, the export phenotype, analysed by RNA FISH, showed nuclear retention of polyadenylated transcripts in induced cells. At this time point cells (+/–Tet) were subjected to a 10-minute pulse of 5-EU, added directly to the culture medium, followed by isolation of the nuclei and RNA extraction. After enrichment and sequencing of the newly synthesised transcripts, we analysed the patterns of transcription. In general, nascent transcription was not perturbed by depletion of TbMex67 (Figure 1B), ruling out that uneven incorporation of 5-EU within the same PTU is related to differential export of transcripts. More striking differences were observed for the TbMex67 RNAi/DN-OE cell line, where we detected a larger number of transcripts that were upregulated. DESeq2 analysis performed on biological triplicates revealed that most upregulated transcripts mapped to chromosomes 6 and 10 (Figure 1C and Supplementary Table S1), particularly the PAGs. The accumulation of PAG mRNAs was previously observed when TbNMD3 was depleted by RNAi (38), suggesting that their degradation is prevented if they do not gain access to the cytoplasm. Indeed, patterns of nascent transcription from exosome RNAi or chromatin-enriched extracts support this notion since they also show accumulation of PAG transcripts (Figure 1D). However, there is a greater increase in PAG transcripts in the TbMex67 RNAi/DN-OE cell lines (Supplementary Figure S1). Moreover, when looking at this region of chromosome 10 (Figure 1D), we observed a clear accumulation of reads downstream of the procyclin PTU that extended over 100 kb, overlapping with the Pol II PTU on the opposite strand (Figures 1D and 2A). The same phenomenon was observed for the procyclin locus on chromosome 6 (Supplementary Figure S2). Overall, all these data demonstrate that, even though PAG reads increase in chromatin extracts and in exosome, TbMex67 or NMD3 RNAi cell lines, the accumulation of transcripts downstream of the PAGs is unique to TbMex67 RNAi/DN-OE, suggesting an additional mode of action for the dominant-negative mutant (Supplementary Figure S1).

### Overexpression of TbMex67 DN results in polymerase I readthrough

Since alignments showed a clear accumulation of transcripts downstream of the PAGs, and these are known to be transcribed by Pol I, we performed Pol I ChIP-seq with TbMex67 RNAi/DN-OE. For these experiments we either used polyclonal antisera directed against the largest subunit of Pol I (RPA1) or a hemagglutinin (HA)-tagged version of this protein (Supplementary Figure S3). In both cases, following Tet induction of TbMex67 RNAi/DN-OE, we detected Pol I enrichment downstream of the PAGs, proceeding as far as the Pol II transcription start site (clearly indicated by the H3K4me3 peaks) on the opposite strand (29) (Figure 2A and Supplementary Figure S2). Changes in Pol I occupancy were also detected at rDNA loci (Supplementary Figure S4A). In this case, the Pol I +/– Tet ratio was reduced over the rDNA genes and increased at their flanking regions, again suggesting a loosening of its boundaries and possible invasion of the rDNA loci by Pol II. Although we did not detect readthrough transcription outside of these loci, we did observe approximately 50% more reads for rRNA in the induced TbMex67 RNAi/DN-OE cell line. However, we cannot tell whether these are the result of higher transcription, including Pol II readthrough, or nuclear accumulation when export is blocked. Indeed, Pol II is enriched at these rDNA loci, although to a minor extent (Supplementary Figure S4B). For global RNA Pol II occupancy, meta-analyses of nascent transcription and ChIP-seq data did not reveal major changes in either initiation or in termination when comparing uninduced and induced cells (Supplementary Figure S5A).

Since these data suggested that chromatin remodeling was occurring, we also performed histone H3 ChIP-seq with the TbMex67 RNAi/DN-OE cell line. In trypanosomes, nucleosomes are known to be partially depleted from regions actively transcribed by Pol I such as the procyclin and ribosomal loci (55,56). Consistent with this, Tet induction resulted in decreased H3 occupancy in these regions where readthrough occurs (Figure 2A and Supplementary Figure S2), but not in other Pol II-transcribed regions such as the tubulin locus on chromosome 1 (Supplementary Figure S5B). The clearance of H3 from these transcription units is not a consequence of lower H3 protein levels, as assessed by western blot analysis (Figure 2B), and the protein is still correctly localised to the nuclear fraction (Figure 2C). These experiments also revealed that, while endogenous wild-type TbMex67 was preferentially localised to the nuclear fraction, more of the DN isoform was in the cytoplasmic fraction (Figure 2C).

Pol I protein levels were also not affected when uninduced and induced TbMex67 RNAi/DN-OE were compared (Figure 2B). However, immunofluorescence experiments indicated that it loses its characteristic horseshoe-shaped nucleolar localisation and invades the Hoechst-positive DNA at the nucleolar periphery. The distribution of the nucleolar marker L1C6 (48,49) indicated that not just Pol I localisation, but also the nucleolar structure was perturbed. L1C6 was found in multiple foci in induced cells, in contrast to the single horseshoe structure in non-induced cells (Figure 2D). Since these data suggested that nucleolar disaggregation and Pol I mislocalisation occur upon TbMex67 RNAi /DN-OE induction, we tested whether these phenomena were comparable to chemical inhibition of Pol I. CX5461 is an inhibitor which is known to cause disintegration of the ESB and nucleolus in bloodstream form trypanosomes (57). Treatment with CX5461 (3 μM for 3 h) also produced nucleolar fragmentation in procyclic forms, reaching 5 L1C6-positive foci per nucleus (Figure 2D and E). In this case, however, Pol I colocalised to discrete foci together with L1C6. This is consistent with different modes of action: CX5461 is likely to be affecting Pol I initiation, while TbMex67 perturbation might be affecting termination.

### The N-terminal domain of TbMex67 is essential and required for nuclear localisation

**Figure 3.**
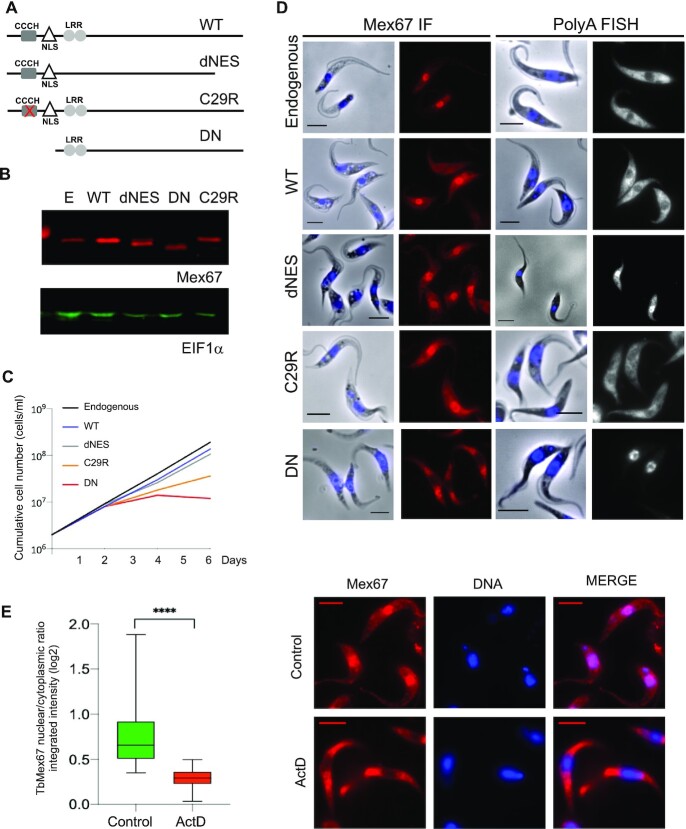
TbMex67 N-terminal domain is essential and required for nuclear localisation. (**A**) Scheme of TbMex67 modular architecture and the different deletion and point mutants (CCCH‐type zinc finger domain (CCCH), leucine‐rich repeats (LRR), nuclear localisation signal (NLS)). (B–D) Western blot (**B**), growth curves (**C**) and immunofluorescence of TbMex67 and polyA RNA FISH (**D**) upon RNAi and complementation with the different variants (scale bar, 5 μm). (**E**) TbMex67 quantification of nuclear/cytoplasmic ratio in the presence of actinomycin D (2 h at 5 μg/ml). Average values are shown with standard deviations and *P*-values, determined using a Mann–Whitney *U* test. Significant *P*-values are indicated by the asterisks above the graphs (****P< 0.0001; ***P< 0.001; **P< 0.01; *P< 0.05). Representative pictures for TbMex67 immunofluorescence from control and actinomycin D treated cells are shown.

To explain the stronger impact of TbMex67 RNAi/DN-OE in comparison to the knockdown alone on transcription, RNA export and cell growth, we took a closer look at the protein's modular architecture (Figure 3A). It transpired that the 37 amino acid deletion in the DN isoform removed not only the CCCH-type zinc finger domain, but also a monopartite nuclear localisation signal (NLS) consensus sequence. To disentangle these two motifs, we took advantage of additional mutants made in the TbMex67 RNAi background. One of these is the TbMex67 C29R mutant, generated by Dostalova and coworkers (33) in which the zinc finger, but not the NLS is disrupted. We also made an additional mutant with a deletion of a portion of the leucine-rich repeat (LRR) that was predicted to be a nuclear export signal. This mutant was named TbMex67 dNES. We monitored over-expression of all these isoforms by western blot analysis (Figure 3B) and assayed for growth phenotypes (Figure 3C). Only those mutants lacking the N-terminal zinc-finger, either by deletion (DN) or point mutation (C29R), showed impaired growth, with DN showing the more profound defect (Figure 3C). We then subjected all these mutants to immunofluorescence assays and corroborated that endogenous TbMex67 is preferentially localised to the nucleus. This was also the case, as expected, for TbMex67 C29R and dNES (Figure 3D). In contrast, however, the DN isoform was distributed throughout the cell, with preference for the cytoplasm (Figure 3D). Consistent with this, polyA RNA FISH showed nuclear accumulation of transcripts upon over-expression of C29R, dNES and DN, being most striking in the latter. This confirms that export is compromised whenever the balance between nuclear and cytoplasmic TbMex67 is perturbed (58).

These results, together with the impact on nascent transcription, stimulated us to evaluate the subcellular localisation of TbMex67 in the presence of the transcriptional inhibitor actinomycin D, which binds DNA and prevents initiation and elongation (59). We found that the nuclear/cytoplasmic ratio decreased significantly in the presence of actinomycin D (Figure 3E), suggesting that, under normal conditions, some TbMex67 is associated with chromatin. To characterise the protein complex involved in active transcription, we performed pulldowns with the largest subunit of Pol II, RPB1. In support of this, TbMex67 was identified in 2 out of 4 pulldowns of RPB1, and in one case this association was disrupted in the presence of actinomycin D (Supplementary Table S2). Multiple nucleoporins (e.g. Nups 62,64,93,98, 109, 155 and 158) were also strongly associated with RPB1 (detected in 4 out of 4 pulldowns) and these associations were lost or reduced following actinomycin D treatment (Supplementary Table S2). In addition, the colocalisation of RPB1 with the nuclear lamin NUP-1 was disrupted by actinomycin D treatment (Supplementary Figure S6). Taken together, these data are consistent with a platform connecting active transcription, RNA processing and export at the nuclear pore.

### TbMex67 association with chromatin depends on its N-terminal domain

**Figure 4.**
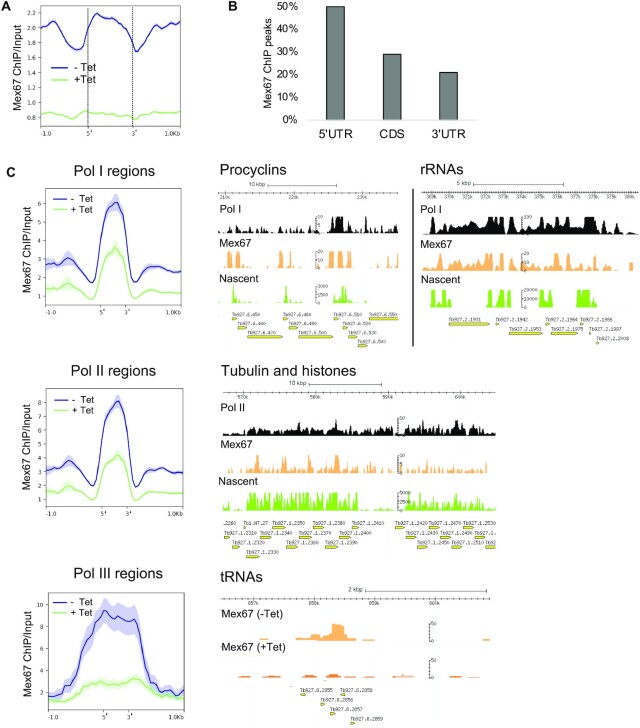
TbMex67 association with chromatin depends on its N-terminal domain. (**A** and **B**) TbMex67 distribution along genes obtained by TbMex67 ChIP-seq. C) Metaplots and mapped reads for TbMex67 ChIP-seq at regions that are specific for each RNA polymerase. Pol I and II ChIP-seq reads (normalised to input), as well as nascent transcripts (RPKM), are also shown. A bed file containing Pol III-transcribed regions was utilised to compensate for the lack of Pol III ChIP. Metaplots show the coverage of TbMex67 ChIP normalised to input from uninduced and induced cells at regions transcribed by each polymerase.

To analyse TbMex67 proximity to chromatin, we performed ChIP-seq with anti-TbMex67 antiserum on uninduced and induced TbMex67 RNAi/DN-OE cells. In uninduced cells, the endogenous protein showed a pattern of occupancy that was reduced at the beginning and end of each gene, known sites of pre-mRNA processing and nucleosome depletion (Figure 4A). Consistent with this, annotation within genes revealed that approximately 50% of the peaks mapped to 5′ untranslated regions (UTRs,) 20% to 3′ UTRs and 30% to coding regions (CDS; Figure 4B). In contrast, ChIP-seq from induced cells showed less enrichment and without clear patterns of distribution along genes (Figure 4A).

When browsing genomic regions transcribed by the different polymerases, TbMex67 ChIP-seq showed occupancy in the procyclin and rDNA loci as well as actively transcribed Pol II genes, such as tubulins and histones, and Pol III genes such as tRNAs (Figure 4C). Occupancy was higher for the Pol I and III loci, suggesting that TbMex67 depletion affects the three polymerases to different extents. Nevertheless, all these regions showed less enrichment in the induced cells. In summary, these analyses confirmed TbMex67’s proximity to genomic DNA.

**Figure 5.**
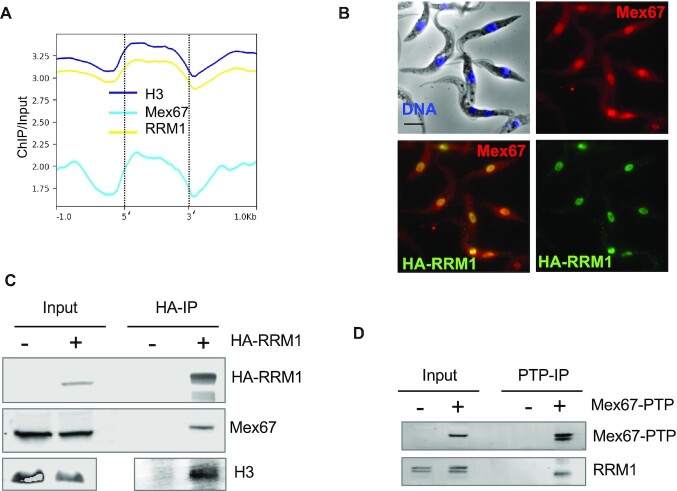
TbMex67 shares genomic occupancy and protein complexes with TbRRM1 and histone H3. (**A**) Genome-wide comparison of TbMex67, histone H3 and TbRRM1 from induced and uninduced cells. (**B**) Immunofluorescence of TbMex67 and HA-TbRRM1 (scale bar, 5 μm). (**C**) Coimmunoprecipitation of TbMex67 and H3 with HA-TbRRM1. (**D**) Immunoprecipitation of TbMex67-PTP.

As shown above, expression of the DN isoform of TbMex67 led to histone depletion downstream of the procyclin loci. When looking at the general distribution of TbMex67 across the genome, we observed a similar pattern to occupancy by H3 and TbRRM1, a modulator of chromatin structure (Figure 5A). To investigate whether these proteins physically interacted with each other, we performed pulldowns with HA-tagged TbRRM1 (51), which has a nuclear distribution that is comparable to that of TbMex67 (Figure 5B). Indeed, TbMex67 and HA-TbRRM1 interact, and in the same pulldown experiment we were also able to detect histone H3 (Figure 5C), as previously reported (51). Conversely, we were able to detect TbRRM1 following immunoprecipitation of PTP-tagged TbMex67 (Figure 5D) (33).

### TbMex67 DN-OE induces accumulation of R-loops and γH2A nuclear foci, and inhibits DNA replication

**Figure 6.**
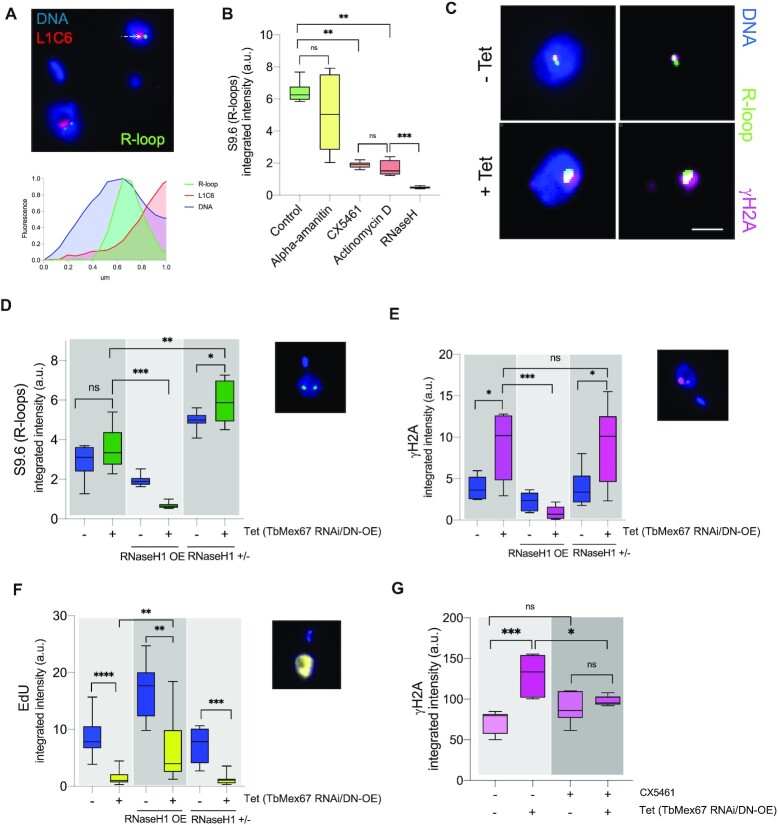
Overexpression of TbMex67 DN induces accumulation of R-loops and γH2A nuclear foci and inhibits DNA replication. (**A**) Visualisation of the nucleolar marker L1C6 and R-loops with quantification of fluorescence intensity across the white arrow. R-loops were detected by staining with the antibody S9.6. (**B**) Quantification of R-loops (S9.6-positive foci), following transcription inhibition by alpha-amanitin (20 h at 100 μg ml^−1^), CX5461 (3 h at 3 μM) and actinomycin D (2 h at 5 μg ml^−1^) or RNase H treatment (20 min at 37ºC). (**C**) Visualisation of R-loops and **γ**H2A in induced (+Tet) and uninduced (-Tet) TbMex67 RNAi/DN-OE cells (Scale bar, 1 μm). (**D**–**F**) Quantification of S9.6-positive foci (D), γH2A (E) and EdU incorporation (F) in induced and uninduced cells. Cell lines over-expressing TbRNaseH1 (OE) or a TbRNaseH1 single knockout (+/-) were used as controls. Representative images are shown. G) Quantification of γH2A-positive loci upon induction of TbMex67 RNAi/DN-OE cells and/or addition of 3 μM CX5461. Average values are shown with standard deviation and p-values, determined using a Mann–Whitney *U* test. Significant p-values are indicated by the asterisks above the graphs (****P< 0.0001; ***P< 0.001; **P< 0.01; *P< 0.05).

Several studies on yeast and mammalian cells have demonstrated that early messenger ribonucleoprotein assembly by the export machinery prevents the formation of detrimental DNA:RNA hybrids (R-loops) that could interfere with Pol II elongation and eventually cause DNA damage and genome instability (60,61). Furthermore, in trypanosomes, R-loops were shown to be enriched at Pol I-transcribed regions, with RNase H enzymes (R-loop resolvers) being required for VSG switching during mammalian host immune evasion (62,63). This led us to speculate that the nuclear accumulation of RNAs upon Tet induction of TbMex67RNAi/DN-OE might be yielding R-loops. To test this hypothesis, we performed immunofluorescence analyses with a commercial antibody which specifically detects R-loops (S9.6). A few perinucleolar foci were detected in uninduced TbMex67RNAi/DN-OE (Figure 6A) and the intensity of staining with S9.6 was reduced when cells were treated with actinomycin D for 2 h prior to fixation or when fixed samples were incubated with the R-loop resolver RNase H1 (Figure 6B). Interestingly, R-loops were also reduced when cells were treated with CX5461 or, although to a lesser extent, by treatment with 100 μg ml^−1^alpha-amanitin (a concentration which inhibits Pol II and Pol III). This suggests that the S9.6 foci observed by microscopy are mostly products of Pol I transcription. Immunoprecipitation with S9.6 followed by high throughput sequencing (DRIP-Seq) confirmed the R-loop distribution already reported for bloodstream form trypanosomes (62) at sites of pre-mRNA processing and nucleosome depletion (Supplementary Figure S7A). Enrichment was highest at Pol I-transcribed genes (rDNA loci and procyclins on chromosome 6), and lower at Pol II- and Pol III-transcribed loci and transcription start sites (Supplementary Figure S7B).

Taking these results into account, we monitored the presence of R-loops by S9.6 staining following induction of TbMex67RNAi/DN-OE. S9.6-positive foci increased in intensity and partially co-localised with γH2A foci (Figure 6C). The latter are often observed in the S and G2 phases of the cell cycle and are indicators of DNA damage (50). To better interpret these results, we generated two additional cell lines in the TbMex67RNAi/DN-OE background. In one cell line, a single allele of TbRNaseH1 was knocked out. Despite repeated attempts, we were unable to generate a TbRNaseH1 null mutant. In the second cell line, TbRNaseH1 was ectopically overexpressed. In both cell lines, we monitored R-loop and γH2A foci as well as ethynyl deoxyuridine (EdU) incorporation, as a measure of DNA replication. Compared to TbMex67RNAi/DN-OE, the TbRNaseH1 single knockout showed increased R-loop and γH2A signals (Figure 6D and E). By contrast, TbRNaseH1 over-expression completely abolished both signals (Figure 6D and E). Interestingly, TbRNaseH1 overexpression allowed the cells to incorporate EdU, although to a lesser extent than uninduced cells (Figure 6F). All these results point to the notion that R-loops are a source of replication stress that is prevented by TbMex67. Indeed, when comparing S9.6 DRIP at TbMex67 peaks, we observed an increase in R-loops when TbMex67RNAi/DN-OE cells are induced (Supplementary Figure S8). Furthermore, RNA Pol I readthrough is an important source of these, since treatment with CX5461 prevented the increase in γH2A nuclear foci observed upon induction of TbMex67RNAi/DN-OE (Figure 6G). In summary, the phenotype observed upon overexpression of the dominant-negative mutant, in contrast to knockdown of TbMex67 alone, is exacerbated by dysregulation of Pol I transcription and an increase in R loop formation. It is also possible that readthrough increases the probability of collisions between the transcription and replication machineries, contributing to DNA damage manifested by γH2A staining.

## DISCUSSION

To analyse the relevance of co-transcriptional export in *Trypanosoma brucei*, we sequenced nascent transcripts from cells in which the general RNA export factor TbMex67 was depleted by RNAi. Even though we did not find many differences between induced and uninduced cells, two regions on chromosomes 6 and 10 showed striking increases in reads downstream of the procyclin genes when TbMex67 was depleted and complemented with a dominant-negative isoform lacking the N-terminal domain. Labelling of nascent transcripts and ChIP-seq experiments revealed that this was the result of Pol I readthrough into the adjacent Pol II PTU, coincident with diminished H3 occupancy in the induced cells. One consequence of this extensive overlapping transcription might be to trigger RNAi and degradation of the mRNAs from the Pol II PTUs. This did not occur, however, suggesting that the transcripts from both strands either failed to form double-stranded RNA or were not accessible to the RNAi machinery.

**Figure 7.**
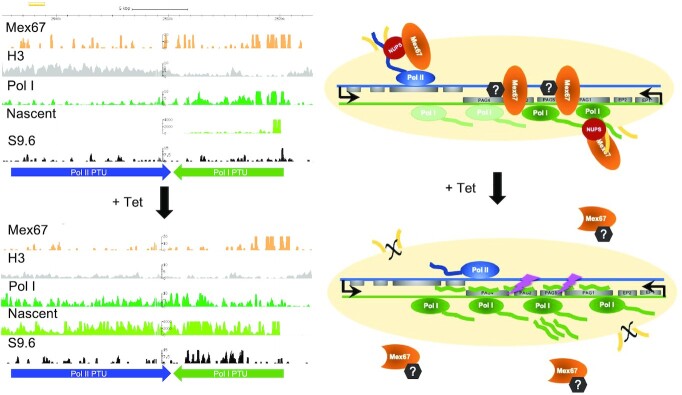
Working model. Mapped reads for nascent transcripts; Pol I, H3 and TbMex67 ChIP-seq and R-loops at the procyclin locus on chromosome 10 from induced (+Tet) and uninduced (–Tet) TbMex67 RNAi/DN-OE cells. R-loops were identified by S9.6 DRIP-seq. Model depicting the role of TbMex67 in preventing readthrough by Pol I. In the absence of the CCCH domain, the protein shows reduced association with chromatin and the nuclear pore and likely sequesters binding partners. NUPS: nucleoporins, Pol I: RNA polymerase I, Pol II: RNA polymerase II. Histone H3 depletion aids Pol I progression. Since the export function of TbMex67 is also impaired, the antisense transcripts retained in the nucleus give rise to R-loops that trigger DNA damage.

To the best of our knowledge, the procyclin loci are unique in being chromosome-internal protein coding genes that are transcribed by RNA Pol I and this may have required trypanosomes to develop specialised processes to regulate them. Although the mechanism by which TbMex67 normally prevents readthrough remains unknown, we showed that it is bound to DNA at the procyclin loci and that this binding decreases in the presence of the dominant-negative isoform. Our laboratory has previously shown that the 5′ UTR of PAG1 contains a region that impedes Pol I transcription, and that deleting it allows transcription to extend further downstream (25). This suggested that proteins binding this region might block progression by Pol I. Figure 7 presents a model of how TbMex67 might attenuate transcription and how co-expression of the dominant-negative mutant would interfere with this. The simplest scenario would be that the wild-type and dominant-negative isoforms compete equally for binding, but only the wild type can impede transcription progression. However, diminished binding when endogenous TbMex67 is depleted, and the mutant isoform is induced (as shown by ChIP-seq in this region, Supplementary Figure S8), argues against this. Wild-type TbMex67 might also recruit other factors that interact with its N-terminal domain, enabling it to bind and terminate transcription, but this would still not explain why concomitant expression of the mutant is more deleterious than RNAi on its own. The more likely explanation is that TbMex67 (independent of its N-terminus) pre-forms a complex with other factors that prevent Pol I progression and that the dominant-negative mutant sequesters this complex away from the procyclin loci. This would have a greater impact than RNAi alone, as the mutant would compete with residual wild-type TbMex67 for these factors. In support of TbMex67 acting as part of a protein complex, a plethora of TbMex67 interactors were recently identified by immunoprecipitation or proximity labelling, and several of these were nucleolar proteins (64), i.e. at the site of Pol I transcription. Given that the dominant-negative mutant lacks the consensus NLS and is predominantly cytoplasmic in cell fractionation experiments, we consider it most likely that the complex is sequestered in the cytoplasm, although we cannot exclude that a proportion of it is associated with the nuclear pore.

The dominant-negative mutant lacks the CCCH domain, which is only present in trypanosomatid Mex67 and has been proposed to fulfil tasks that are performed by additional adaptor proteins in other eukaryotes, particularly given that TbMex67 lacks an RRM domain. We have shown that the CCCH domain is important for nuclear localisation of TbMex67 and its recruitment to chromatin, as analysed by ChIP. However, it is also possible that this domain binds RNA, which could account for TbMex67’s proximity to transcription sites. Alternatively, this role could be fulfilled through its interaction with TbRRM1, which is also known to bind RNA (51), or other RNA-binding proteins associated with TbMex67 (64). It is also worth noting that the monopartite NLS consensus sequence found in full-length TbMex67, but absent from the dominant-negative mutant, could serve as a DNA-binding domain. This has been shown for several proteins in other organisms, where NLS motifs co-localised with DNA (but not RNA)-binding regions (65).

The finding that readthrough does not occur from the rDNA loci implies that, in contrast to the procyclin transcription units, these are insulated by factors that are independent of TbMex67. As far as Pol II transcription is concerned, meta-analyses of nascent transcription and Pol II occupancy of divergent and head-to-tail transcription initiation sites did not show major changes in initiation or termination upon depletion of TbMex67 and overexpression of the dominant negative mutant. This suggests that TbMex67 is also not required to insulate transcription between Pol II PTUs, and that its main or sole function in this context is to facilitate co-transcriptional export. The proximity of Pol II to the nuclear pore is confirmed by our finding that its largest subunit is strongly associated with nucleoporins, and that blocking transcription disrupts this association. It is also consistent with data showing that TbMex67 and TbNup158 not only interact with each other (32) and with RPB1 (this study), but also with nuclear RNA processing factors (64) and chromatin-associated proteins such as TbRRM1 (this study) and FACT subunit POB3 and bromodomain proteins (64).

In terms of its function in RNA export, and its more widespread association with chromatin, it is tempting to speculate that TbMex67 ‘travels’ with the RNA polymerase(s) and that, at sites of pre-mRNA processing, the whole newly assembled ribonucleoprotein (RNP) complex is targeted to and through the nuclear pore. TbMex67’s interactions with nucleoporins and Ran proteins support this hypothesis (32). Similar studies assessing Mex67 ChIP were performed in yeast by Dieppois and coworkers (66). They proposed that co-transcriptional binding of Mex67p is RNA-independent and may contribute to gene anchoring to the nuclear pore by interacting with activated chromatin rather than nascent RNA. It is worth noting, however, that yeast Mex67, which lacks the CCCH domain, is believed to bind RNA poorly and functions instead as a mobile nucleoporin (67). When assessed for binding to the GAL10 gene, yeast Mex67p showed a pattern comparable to our TbMex67 ChIP-seq metaplot: it was detected at very low levels at the promoter, increasing at the 5′ UTR, and reaching a maximum in the coding sequence, before declining towards the 3′ UTR (66). Furthermore, Zander *et al.* showed yeast Mex67 interacted with Pol II upon heat stress, probably aided by the transcription factor HSF1 (68). Our study also revealed the presence of TbMex67 at Pol I-transcribed regions of the genome. This is not surprising since procyclin transcripts are processed as mRNAs and therefore might assemble comparable mRNPs. In addition, procyclins are transcribed at the same perinucleolar location as rRNAs, which have been reported to bind TbMex67 in trypanosomes (37). Also linking TbMex67 to Pol I transcription, we observed disintegration of the nucleolus when TbMex67 was depleted by RNAi and the dominant negative isoform was overexpressed. Of note, procyclin and VSG genes exhibited higher expression when the nuclear lamina proteins NUP-1 and NUP-2 were down-regulated by RNAi, linking the regulation of Pol I transcription to the nuclear envelope and nuclear pore complexes (69,70).

All the aforementioned interactions place TbMex67 at multiple layers of RNA biogenesis and lead us to speculate that it could also aid genome integrity, as was reported for other export factors in mammalian cells (71). Indeed, mutations in genes involved in pre-mRNA processing and in the biogenesis and export of mRNPs induce DNA damage and genome instability, frequently mediated by R-loops (71). The 5′ and 3′ ends of genes were previously reported to be sites of enrichment for R-loops in trypanosomes, which was suggested to be related to mRNA processing (62). Our S9.6 DRIP analysis confirmed this enrichment, with a slight preference for 5′ ends. These reports also showed that the distribution of R-loops along genes was inversely related to nucleosome occupancy, which we also found to be the case for TbMex67. If one of TbMex67’s functions is to prevent R-loop formation, depleting it should increase their frequency, and this what we observed following induction of TbMex67 RNAi/DN-OE. Even though we did not observe new DNA:RNA hybrids in the genome, we detected an increase both by immunofluorescence and DRIP meta-analysis. This suggests that TbMex67 could prevent the formation of potentially damaging R-loops by helping release the newly synthesised RNA from its template and favoring mRNA export. Pol I-transcribed genes seem to be R-loop hotspots, which was already reported for bloodstream forms, where they were found not only on rRNA and VSG genes, but also at procyclin loci (63). It is likely that the high rates of transcription of these regions increases the chance of nascent RNA still being associated with the genome. Under normal circumstances this does not appear to be harmful for DNA replication and cell cycle progression. However, upon depletion of TbMex67 and overexpression of the dominant mutant, deregulation of Pol I transcription seems to be an important source of detrimental R-loop formation and DNA damage since its inhibition by CX5461 prevents an increase in γH2A. It is also possible that readthrough Pol I collides with DNA polymerases from nearby replication origins, which have been mapped to the boundaries of the transcription units by replication initiator ORC1/CDC6 genomic occupancy (72).

In summary (Figure 7), our results suggest that, in addition to its function in RNA export, TbMex67 associates with chromatin where it has two important impacts on Pol I transcription. The first is that it prevents readthrough beyond the procyclin loci and, as a consequence, protects against excessive R-loop formation. This, in turn, could safeguard the cell against Pol I-induced replication stress and possible DNA damage.

## DATA AVAILABILITY

European Nucleotide Archives (ENA) http://www.ebi.ac.uk/ena as study PRJEB57072. Proteomics data have been deposited in the Proteomics Identification Database (PRIDE), under accession code PXD039878.

## Supplementary Material

gkad251_Supplemental_Files

## Notes

Present address: Berta Pozzi, CONICET-Universidad de Buenos Aires, Instituto de Fisiología, Biología Molecular y Neurociencias (IFIBYNE), Buenos Aires, Argentina.

Present address: Arunasalam Naguleswaran, Institute of Animal Pathology, Vetsuisse Faculty, University of Bern, Bern, Switzerland.

Present address: Francesca Florini, Department of Microbiology and Immunology, Weill Cornell Medical College, New York, NY, USA.
