# Supplementary material for: The RNA export factor TbMex67 connects transcription and RNA export in Trypanosoma brucei and sets boundaries for RNA polymerase I
Source: Nucleic Acids Res. 2023 Apr 18;51(10):5177–92. doi: 10.1093/nar/gkad251 (PMC10250216; doi:10.1093/nar/gkad251)
Supplement: gkad251_Supplemental_Files [file gkad251_supplemental_files.zip › Supp material_3.pdf]

Supplementary information to:

The RNA export factor TbMex67 connects transcription and RNA export in *Trypanosoma brucei* and sets boundaries for RNA polymerase I.

Berta Pozzi[1*,2], Arunasalam Naguleswaran[1,3], Francesca Florini[1,4], Zahra Rezaei[5] and Isabel Roditi[1,*]

[1] Institute of Cell Biology, University of Bern, Bern, Switzerland

[2] Present address: CONICET-Universidad de Buenos Aires, Instituto de Fisiología, Biología Molecular y Neurociencias (IFIBYNE), Buenos Aires, Argentina.

[3] Present address: Institute of Animal Pathology, Vetsuisse Faculty, University of Bern, Bern, Switzerland.

[4] Present address: Department of Microbiology and Immunology, Weill Cornell Medical College, New York, NY, United States.

[5] Professor Alborzi Clinical Microbiology Research Center, Shiraz University of Medical Sciences, Shiraz, Iran.

* Corresponding authors: bertapozzi@fbmc.fcen.uba.ar; isabel.roditi@unibe.ch

Figure S1

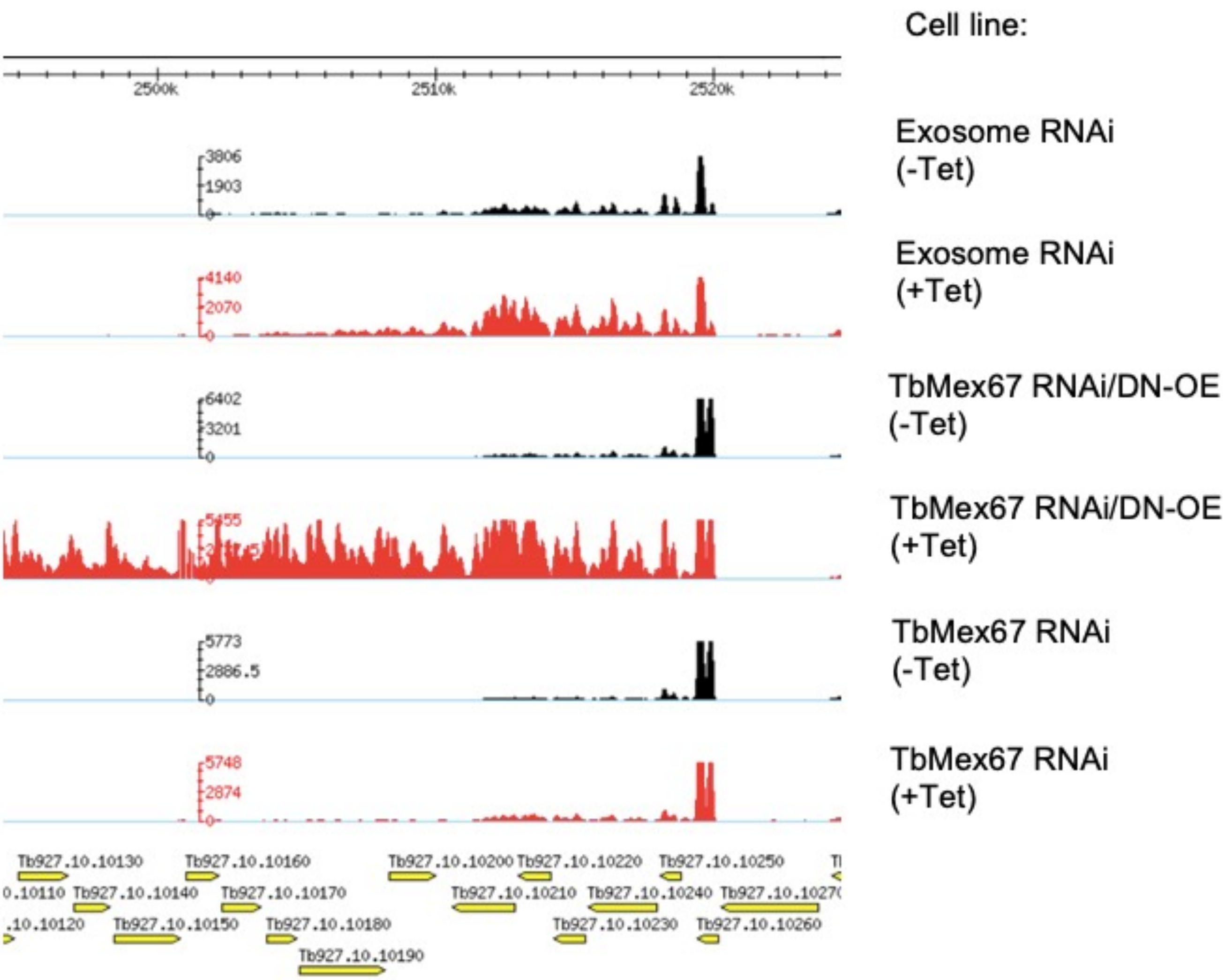

Supplementary figure 1. Patterns of nascent transcription mapped reads visualised in Genome Browser for a region in chromosome 10 where procyclins and PAGs are encoded. RPKM for split reverse strand for induced (+Tet) and non-induced (-Tet) cell lines are shown. Tracks are scaled to the local minimum/maximum.

Figure S2

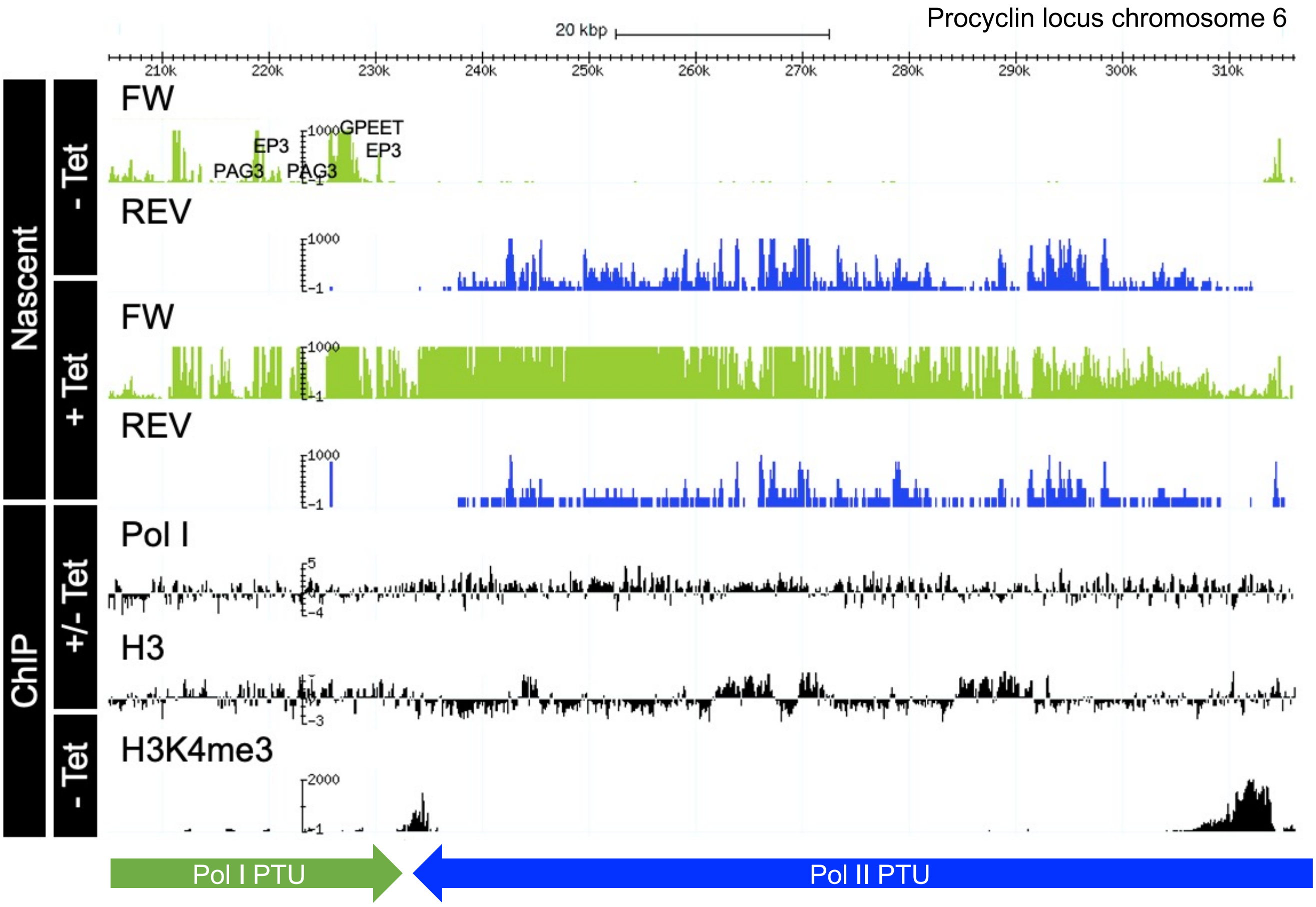

Supplementary figure 2. Patterns of nascent transcription and Pol I and H3 ChIP-seq mapped reads visualised in Genome Browser for a region in chromosome 6 where procyclins and PAGs are encoded. In the case of nascent transcripts, RPKM for split forward and reverse strands for induced (+Tet) and non-induced (-Tet) TbMex67 RNAi/DN-OE are shown. For ChIP-seq of Pol I and H3, only the ratios of induced to non-induced reads are shown (+/- Tet) using a log2 scale. The track for H3K4me3 ChIP-seq from uninduced cells is shown as an indicator of transcription start sites (Wright 2010) . The green tracks and arrow show the directionality of the Pol I PTU (polycistronic transcription unit) while the blue tracks and arrow show the directionality of the Pol II PTU.

Figure S3

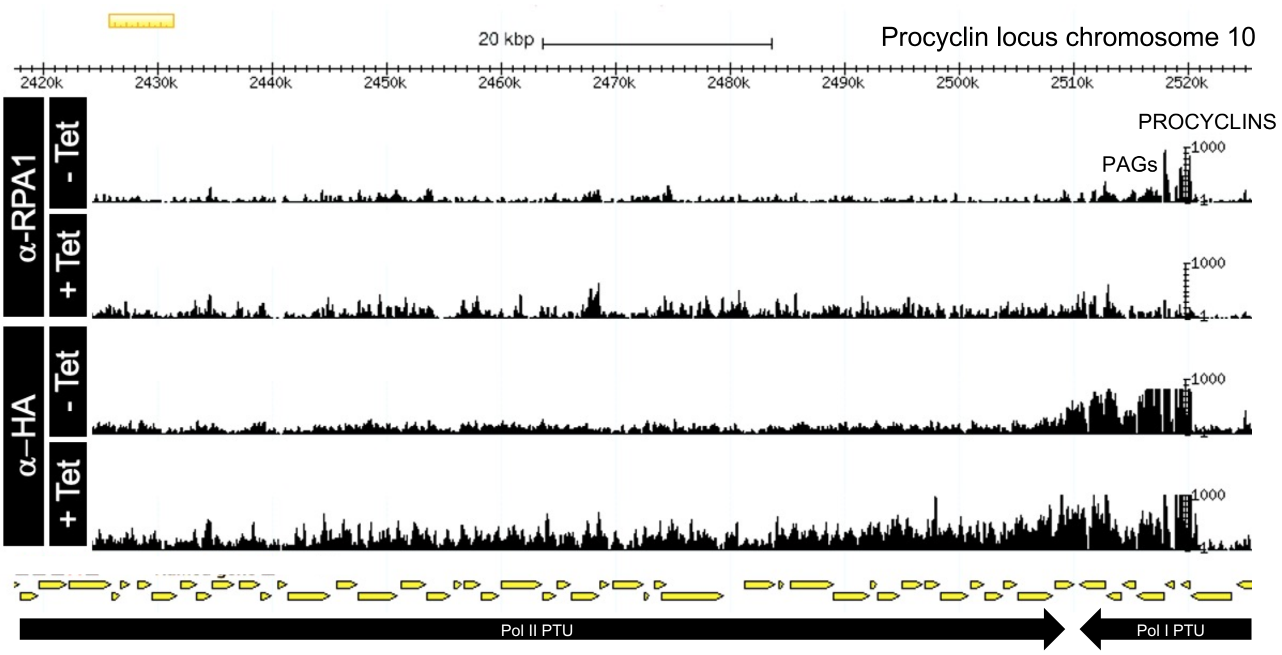

Supplementary figure 3. Mapped reads for Pol I ChIP-seq at the procyclin locus on chromosome 10 from induced and uninduced TbMex67 RNAi/DN-OE cells. ChIP was perfomed either with anti-RPA1 antibody or anti-HA from a cell line with *in situ* tagged RPA1, as indicated.

Figure S4

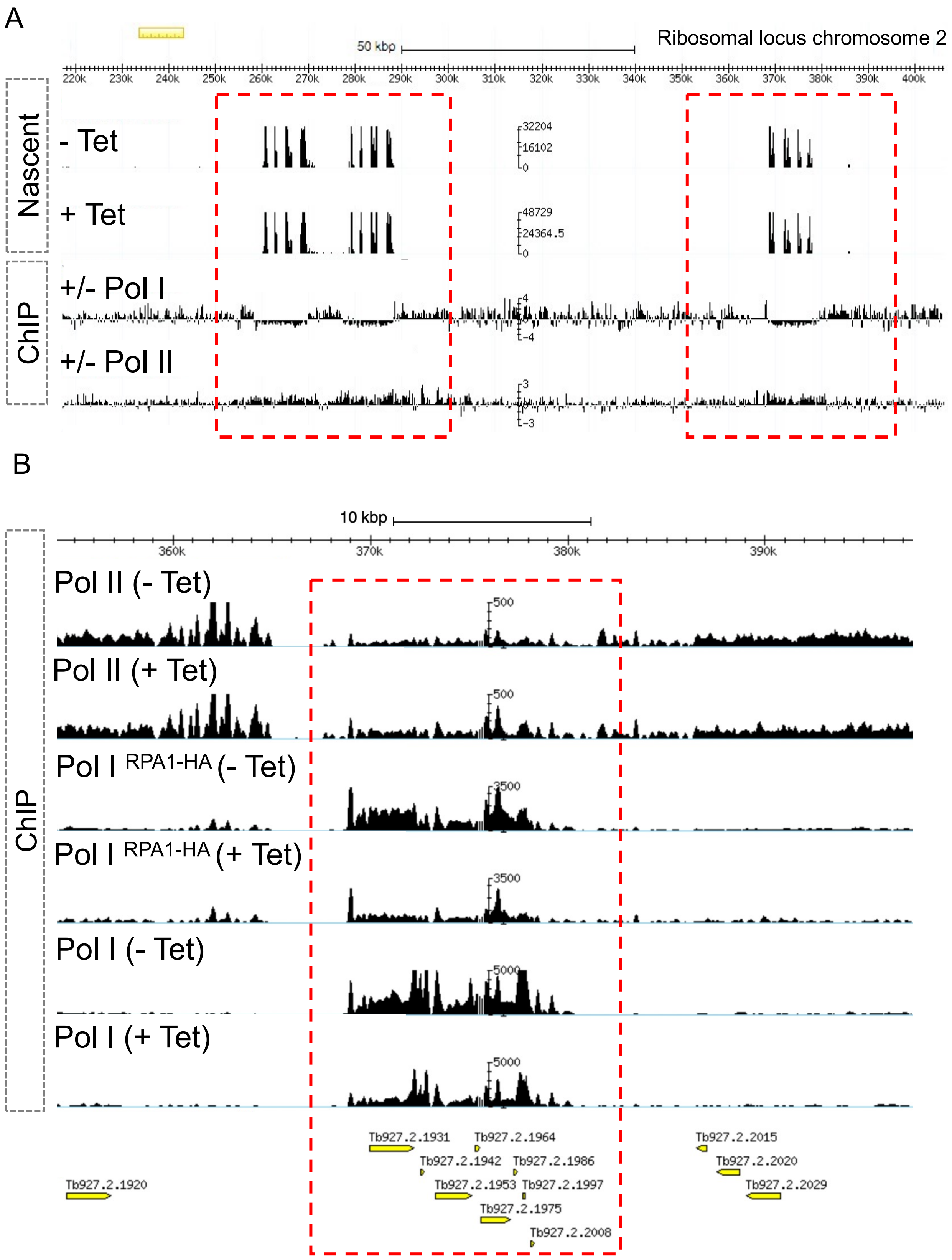

Supplementary figure 4. Mapped reads for nascent transcription and Pol I and Pol II ChIP-seq from induced and uninduced TbMex67 RNAi/DN-OE cells at a ribosomal locus on chromosome 2 (red boxes). In panel A, ChIPs are presented as +/- Tet ratio (log2 scale). In panel B, tracks for induced and uninduced cells are shown separately for both Pol I and II ChIP-seq (RPKM scale). For Pol I, ChIP-seq from pulled-down RPA1-HA is also shown.

Figure S5

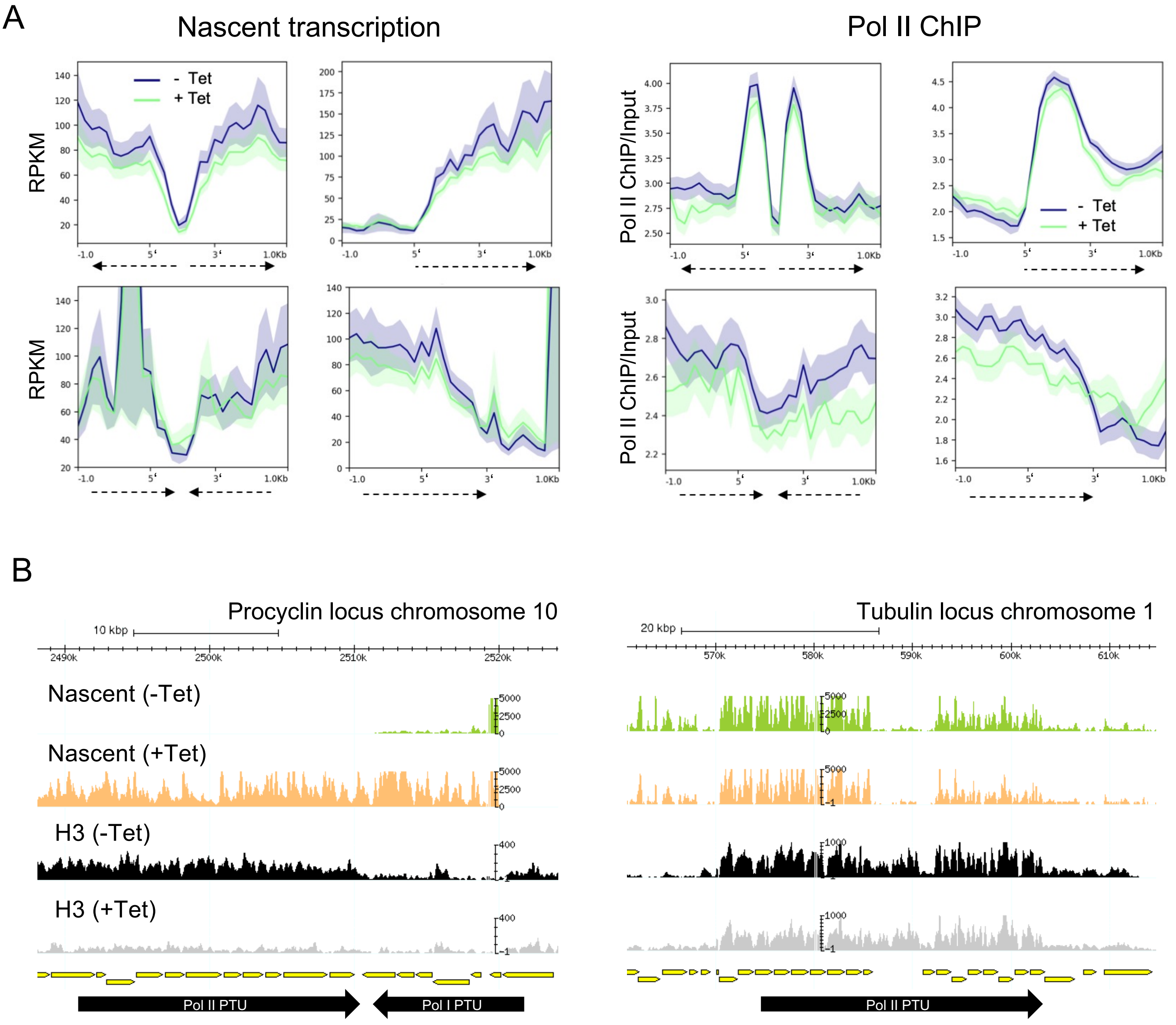

Supplementary figure 5

A) Metaplots for nascent transcription and Pol II ChIP-seq from induced (+Tet) and uninduced (-Tet) TbMex67 RNAi/DN-OE cells are shown at regions of divergent and head-to-tail transcription initiation as well as convergent and head-to-tail transcription termination.

B) Mapped reads for nascent transcription as well as histone H3 ChIP-seq at the procyclin locus on chromosome 10 and the tubulin locus in chromosome 1 from induced (+Tet) and uninduced (-Tet) TbMex67 RNAi/DN-OE cells.

Figure S6

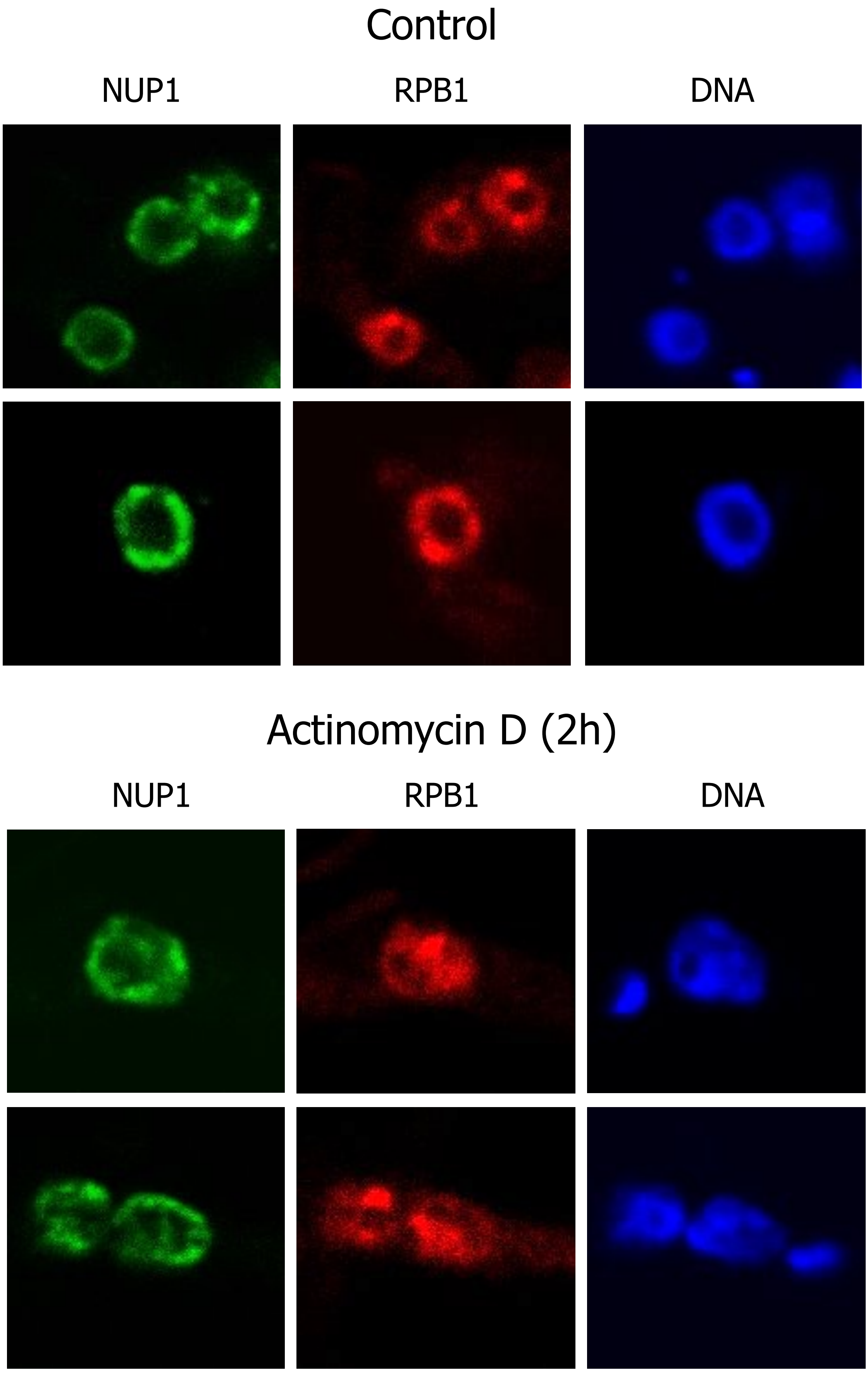

Supplementary figure 6. Immunofluorescence of NUP1 and RPB1 from procyclic forms of EATRO1125 under control conditions (DMSO) and treatment with actinomycin D (2 h at 5 µg/ml). DNA was stained with Hoechst dye.

Figure S7

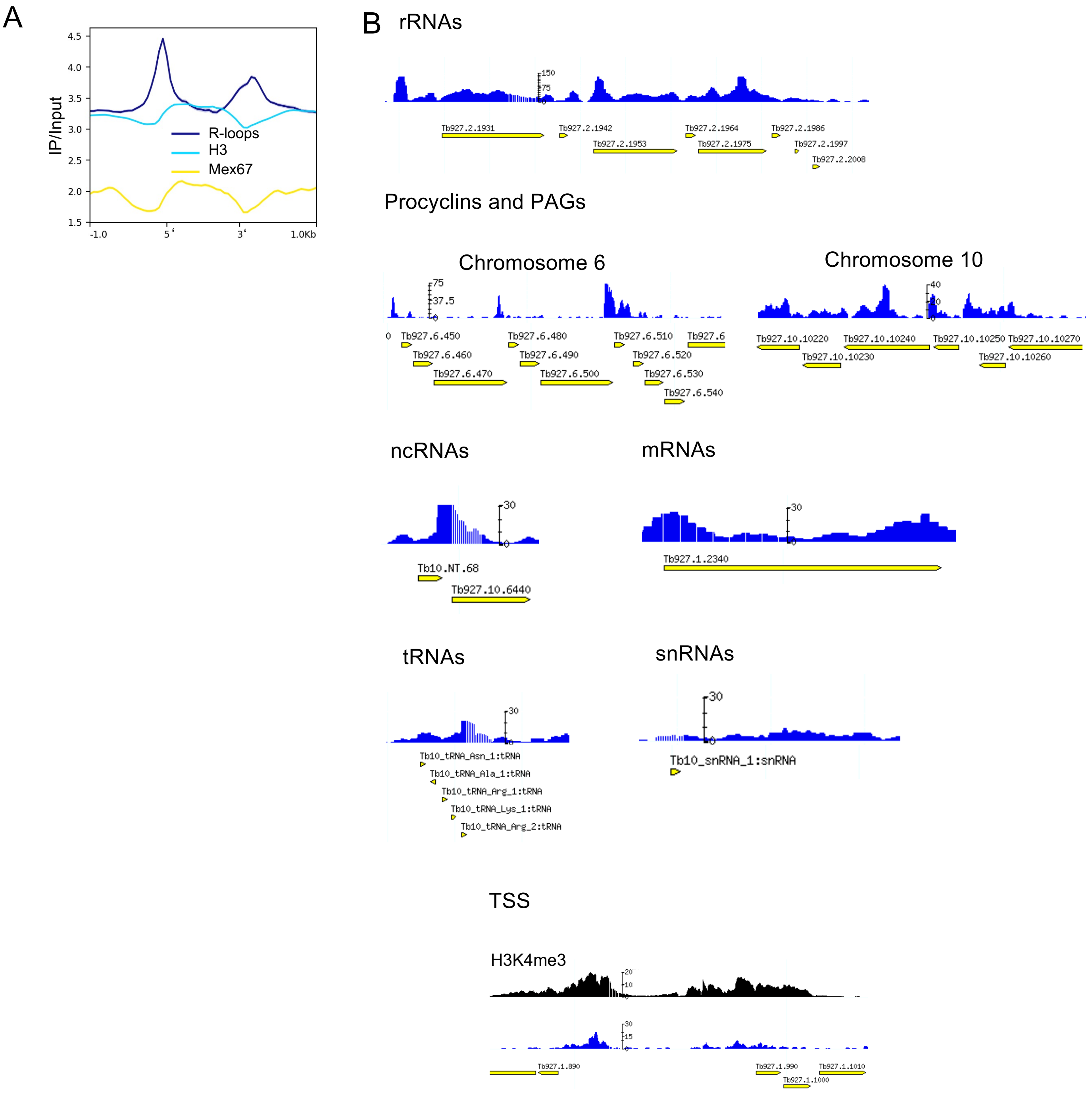

Supplementary figure 7

A) Distribution of R-loops, histone H3 and TbMex67 along genes. R-loops were immunoprecipitated with S9.6 and subjected to DRIP-seq.

B) Examples of R-loops in loci transcribed by Pol I (rRNAs, procyclins and PAGs), Pol II (mRNA, snRNA, ncRNA) and and Pol III (tRNA). TSS: transcription start sites (data from Siegel et al., 2009)

Figure S8

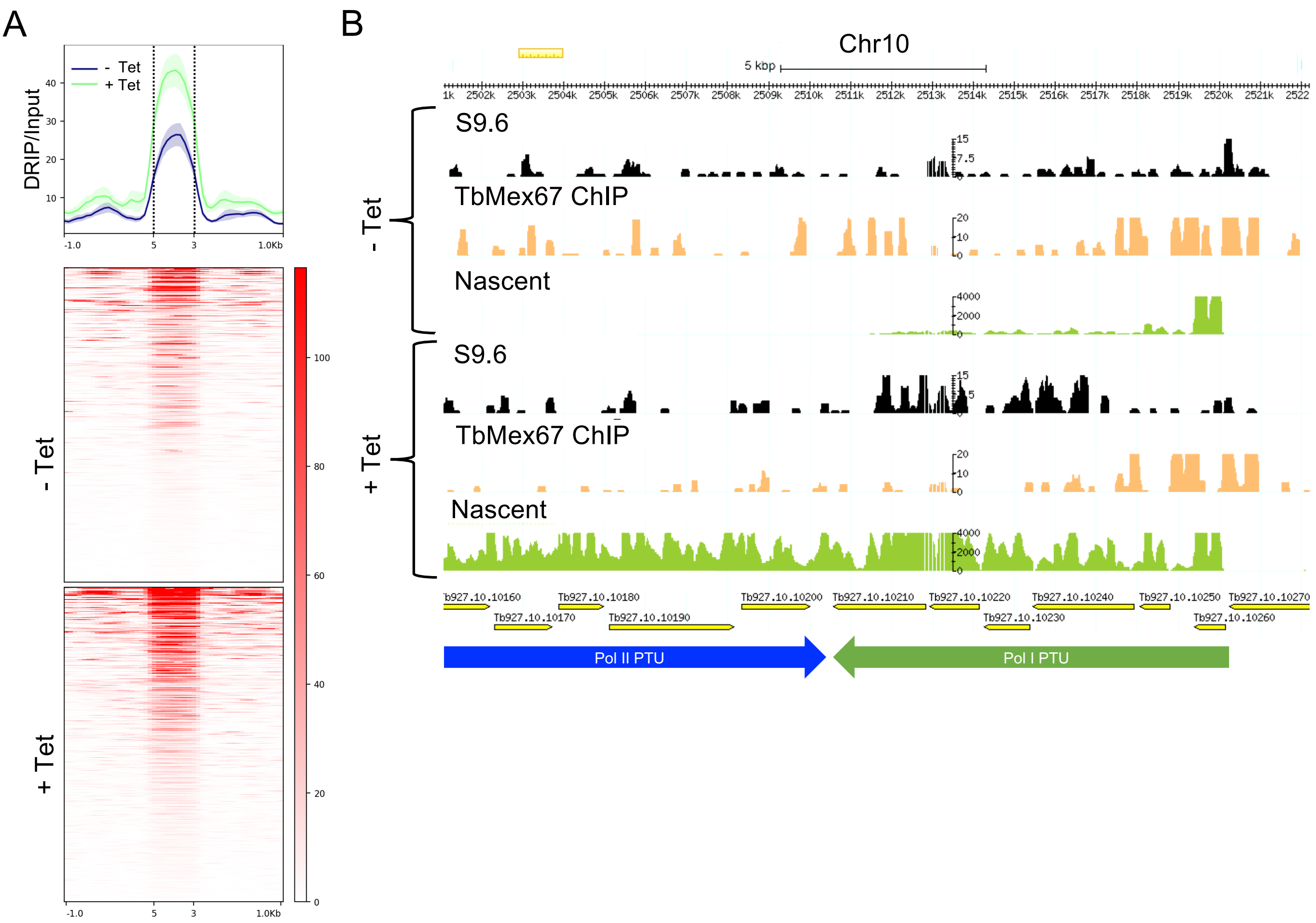

Supplementary figure 8

A) Metaplot and heat map of R-loop enrichment along TbMex67 peaks from induced (+Tet) and uninduced (-Tet) cells. Experiments were performed with uninduced (-Tet) or induced (+ Tet) TbMex67 RNAi/DN-OE.

B) Mapped reads for S9.6 DRIP-seq, TbMex67 ChIP-seq as wells as nascent transcription at the procyclin locus on chromosome 10 from induced (+Tet) and uninduced (-Tet) TbMex67 RNAi/DN-OE cells.

Table S3: List of oligonucleotides

Oligo	Sequence
**Upstream FW KO Tb427.07.4930**	5'-CGA ATG GAT TCA CTT CGT TAG GGT GCA CCA GTA TAA TGC AGA CCT GCT GC-3'
**Downstream REV KO Tb427.07.4930**	5'-CCA CAG AGA ATC ACT TGC GTC ATG TAA CCA CCG GAA CCA CTA CCA GAA CC-3'
**5' sgRNA primer KO Tb427.07.4930**	5'-GAA ATT AAT ACG ACT CAC TAT AGG AAA AGC GAG CAG AGA AGG CGG TTT TAG AGC TAG AAA TAG C-3'
**3' sgRNA primer KO Tb427.07.4930**	5'-GAA ATT AAT ACG ACT CAC TAT AGG ACA GGA ACG CCA TCA CTG ATG TTT TAG AGC TAG AAA TAG C-3'
**FW RNAseH1 pLEW111**	5'-GGG AAG CTT ATG GGA AAG AAA AGG TTT TA-3'
**REV RNAseH1 pLEW111**	5'-TAG GAT CCT TAG CCA AAG GGC GAT TCA C-3'
**FW screen RNAseH1 KO**	5'-GCC GAA GAC TAG TGC AAG CC-3'
**Mex67 dNES 73 FW**	5'-GAA GCC AAC CTA ATC AAG TCG GTT GAG TCA TTG CAA GAG TTG-3'
**Mex67 dNES REV**	5'-CTG CAT CGC TAG ATG TGA CAC CGA CAA TAT GCC ATT GCC-3'
**Tb927.8.5090 C-tag FW**	5'-TTG CGG GAT GTG CTC GAG AGG AAC TTT GCA GGT TCT GGT AGT GGT TCC GG-3'
**Tb927.8.5090 C-tag REV**	5'-AAA ACA AAA AAG GTA ATA TAC ATA TAT ATA CCA ATT TGA GAG ACC TGT GC-3'
**Tb927.8.5090 C-tag 3'gRNA**	5'-GAA ATT AAT ACG ACT CAC TAT AGG TAT ACT TAA AAT AGC GGT TCG TTT TAG AGC TAG AAA TAG C-3'

Table S4: List of antibodies

Antibody	Dilution	Kindly provided by	References
**α-RPA1 Monoclonal**	IF: 1/100<br>WB:1/1000<br>ChIP: 5 ug/IP	Miguel Navarro	(Navarro et al., 2007)
**α-RPB1**	IF: 1/100<br>WB:1/2000<br>ChIP: 5 ug/IP<br>IP: 5 ug/IP	Vivian Bellofatto	(Das and Bellofatto, 2006; Florini et al., 2019)
**α-NUP1**	IF: 1/1000	Klaus Ersfeld	(Ersfeld, 2011)
**α-Mex67**	IF: 1/100<br>WB:1/2000	Mark Carrington	(Dostalova et al., 2013)
**α-H3**	WB:1/5000<br>ChIP: 2 ug/IP	Abcam ab1791	(Naguleswaran et al., 2015)
**α-HA**	IF: 1/100<br>WB:1/2500<br>IP: 2 ug	Sigma-Aldrich (3F10)	(Florini et al., 2019; Naguleswaran et al., 2015)
**α-EIF1a**	WB:1/10000	Santa Cruz	(Dostalova et al., 2013)
**L1C6**	IF: 1/100	Keith Gull	(Devaux et al., 2007; Schumann Burkard et al., 2013)
**S9.6**	IF: 1/500<br>DRIP: 1ug/IP	Kerafast	(Briggs et al., 2018)
**α-RRM1**	WB:1/5000	John Boothroyd	(Manger and Boothroyd, 1998)
**α-TbγH2A**	IF: 1/100	David Horn	(Glover and Horn, 2012)
**α-rabbit Cy3<br>α-mouse Alexa 488<br>α-mouse Alexa 647**	IF: 1/2000	Thermo Fisher	(Bevkal et al., 2021; Florini et al., 2019; Naguleswaran et al., 2015)

Bevkal S, Naguleswaran A, Rehmann R, Kaiser M, Heller M, Roditi I. 2021. An Alba-domain protein required for proteome remodelling during trypanosome differentiation and host transition. *PLoS Pathog*. doi:10.1371/journal.ppat.1009239

Briggs E, Hamilton G, Crouch K, Lapsley C, McCulloch R. 2018. Genome-wide mapping reveals conserved and diverged R-loop activities in the unusual genetic landscape of the African trypanosome genome. *Nucleic Acids Res*. doi:10.1093/nar/gky928

Das A, Li H, Liu T, Bellofatto V. 2006. Biochemical characterization of Trypanosoma brucei RNA polymerase II. *Mol Biochem Parasitol*. doi:10.1016/j.molbiopara.2006.08.002

Devaux S, Kelly S, Lecordier L, Wickstead B, Perez-Morga D, Pays E, Vanhamme L, Gull K. 2007. Diversification of function by different isoforms of conventionally shared RNA polymerase subunits. *Mol Biol Cell*. doi:10.1091/mbc.E06-09-0841

Dostalova A, Käser S, Cristodero M, Schimanski B. 2013. The nuclear mRNA export receptor Mex67-Mtr2 of Trypanosoma brucei contains a unique and essential zinc finger motif. *Mol Microbiol*. doi:10.1111/mmi.12217

Ersfeld K. 2011. Nuclear architecture, genome and chromatin organisation in Trypanosoma brucei. *Res Microbiol*. doi:10.1016/j.resmic.2011.01.014

Florini F, Naguleswaran A, Gharib WH, Bringaud F, Roditi I. 2019. Unexpected diversity in eukaryotic transcription revealed by the retrotransposon hotspot family of Trypanosoma brucei. *Nucleic Acids Res*. doi:10.1093/nar/gky1255

Glover L, Horn D. 2012. Trypanosomal histone γh2A and the DNA damage response. *Mol Biochem Parasitol*. doi:10.1016/j.molbiopara.2012.01.008

Manger ID, Boothroyd JC. 1998. Identification of a nuclear protein in Trypanosoma brucei with homology to RNA-binding proteins from cis-splicing systems. *Mol Biochem Parasitol*. doi:10.1016/S0166-6851(98)00118-2

Naguleswaran A, Gunasekera K, Schimanski B, Heller M, Hemphill A, Ochsenreiter T, Roditi I. 2015. Trypanosoma brucei RRM1 is a nuclear RNA-binding protein and modulator of chromatin structure. *MBio*. doi:10.1128/mBio.00114-15

Navarro M, Peñate X, Landeira D. 2007. Nuclear architecture underlying gene expression in Trypanosoma brucei. *Trends Microbiol*. doi:10.1016/j.tim.2007.04.004

Schumann Burkard G, Käser S, de Araújo PR, Schimanski B, Naguleswaran A, Knüsel S, Heller M, Roditi I. 2013. Nucleolar proteins regulate stage-specific gene expression and ribosomal RNA maturation in Trypanosoma brucei. *Mol Microbiol*. doi:10.1111/mmi.12227
